# Supplementary material for: A Comprehensive Assessment of Ultraviolet-Radiation-Induced Mutations in Flammulina filiformis Using Whole-Genome Resequencing
Source: J Fungi (Basel). 2024 Mar 20;10(3):228. doi: 10.3390/jof10030228 (PMC10971301; doi:10.3390/jof10030228)
Supplement: Supplementary file 1 [file jof-10-00228-s001.zip › Supplementary Material S8/KEGG annotation/out/64381550635650.os/KO/out_map/map05132.html]

KEGG PATHWAY: Salmonella infection - Reference pathway


|  |  |
| --- | --- |
| **Salmonella infection - Reference pathway** |  |

[
Pathway menu
| Organism menu
| Pathway entry
| Show description
| User data mapping
]

Salmonella infection usually presents as a self-limiting gastroenteritis or the more severe typhoid fever and bacteremia. The common disease-causing Salmonella species in human is a single species, Salmonella enterica, which has numerous serovars.
Following intestinal colonization Salmonella inject effector proteins into the host cells using a type III secretion system (T3SS), T3SS1. Then a small group of effector proteins induce rearrangement of the actin cytoskeleton resulting in membrane ruffles and rapid internalization of the bacteria.The T3SS2 is responsible for translocating effector proteins that direct Salmonella-containing vacuole (SCV) maturation. The majority of the bacteria are known to survive and replicate in SCV.

|  |  |
| --- | --- |
| Reference pathway | 100% |
